# Uncovering the Molecular Underpinnings of Oxidative Stress-Induced Senescence

**DOI:** 10.1093/geroni/igaa057.2656

**Published:** 2020-12-16

**Authors:** Melissa Carpenter, Laura Corrales-Diaz Pomatto, Jonathan Kato, Sarah Wong, Oye Bosompra, Michel Bernier, Rafael de Cabo

**Affiliations:** National Institute on Aging, Bethesda, Maryland, United States

## Abstract

The aging process is sexually dimorphic, with males having higher occurrence rates of cancer and facing a greater risk of mortality. Sexual dimorphism in the response to cellular damage may account for distinct phenotypic changes with age as they relate to the accumulation of cellular damage leading to cancer. Cellular senescence triggers permanent cell cycle arrest in order to protect against malignant growth. However, organismal senescence increases with age and is associated with the release of pro-inflammatory signals (cytokines, chemokines, and proteases) known as the ‘senescence-associated-secretory-phenotype’ (SASP) that, if unchecked, accelerates tissue damage and creates a microenvironment ripe for cancer development. In this study, we hypothesized that sexual disparities in mortality and cancer prevalence stems from differences in the rate of accumulation of senescent cells in mice. Male and female C57BL/6J mice were fed ad libitum or subjected to 30% calorie restriction, a nutritional intervention known to delay the onset of various cancers and prevent senescent cell accumulation. Primary skin fibroblasts were collected longitudinally to allow measurement of cell proliferation, wound healing and the release of SASP factors. The results indicate that when compared to males, fibroblasts of CR-fed females showed significant improvements in cell growth rate, wound healing and SASP markers vs. AL controls. Work is underway to determine how sex influences cellular protective pathways. Thus, like other cell processes, cellular senescence is unequal between males and females and CR delays the emergence of the senescence phenotype.

